# Congenital mid-ureteral stricture: a case report of two patients

**DOI:** 10.1186/s12894-018-0423-7

**Published:** 2018-11-26

**Authors:** Hamdan Alhazmi, Abdullah Fouda Neel

**Affiliations:** Division of Urology, Department of Surgery, College of Medicine and King Saud University Medical City, King Saud University, PO Box 7805, Riyadh, 11472 Kingdom of Saudi Arabia

**Keywords:** Mid-ureteral stricture, Children, Antenatal hydronephrosis, Ureter

## Abstract

**Background:**

Congenital hydronephrosis is a common foetal anomaly. There are numerous causes of hydronephrosis. The diagnosis of ureteral anomalies remains challenging. Congenital mid-ureteral stricture (CMS) is less common than proximal and distal strictures. In most cases involving CMS, this condition is diagnosed intra-operatively. The gold standard treatment is resection of the stenosed segment and ureteroureterostomy.

**Case presentation:**

We report two patients with CMS which presented as antenatal hydronephrosis with postnatal workup showed a picture of pelviuretric junction obstruction which required surgical correction. Intraoperative retrograde pyelography (RGP) confirmed the diagnosis of mid ureteral stricture which make us to change the planned surgical intervention from pyeloplasty to excision of the ureteral stricture and ureteroureterostomy as definitive management.

**Conclusion:**

CMS should be considered whenever proximal mega-ureter is an associated finding. Despite advanced radiological modalities, RGP remains the mainstay approach for diagnosing ureteral anomalies.

## Background

Congenital ureteral stricture is a rare cause of paediatric hydronephrosis [1]. Congenital mid-ureteral stricture (CMS) is associated with severe hydronephrosis and proximal ureteral dilatation [2, 3]. However, this condition is typically diagnosed intra-operatively [3]. Here, we report two cases of CMS that were managed at our institute.

## Case presentation

### Case 1

**Fig. 1:**
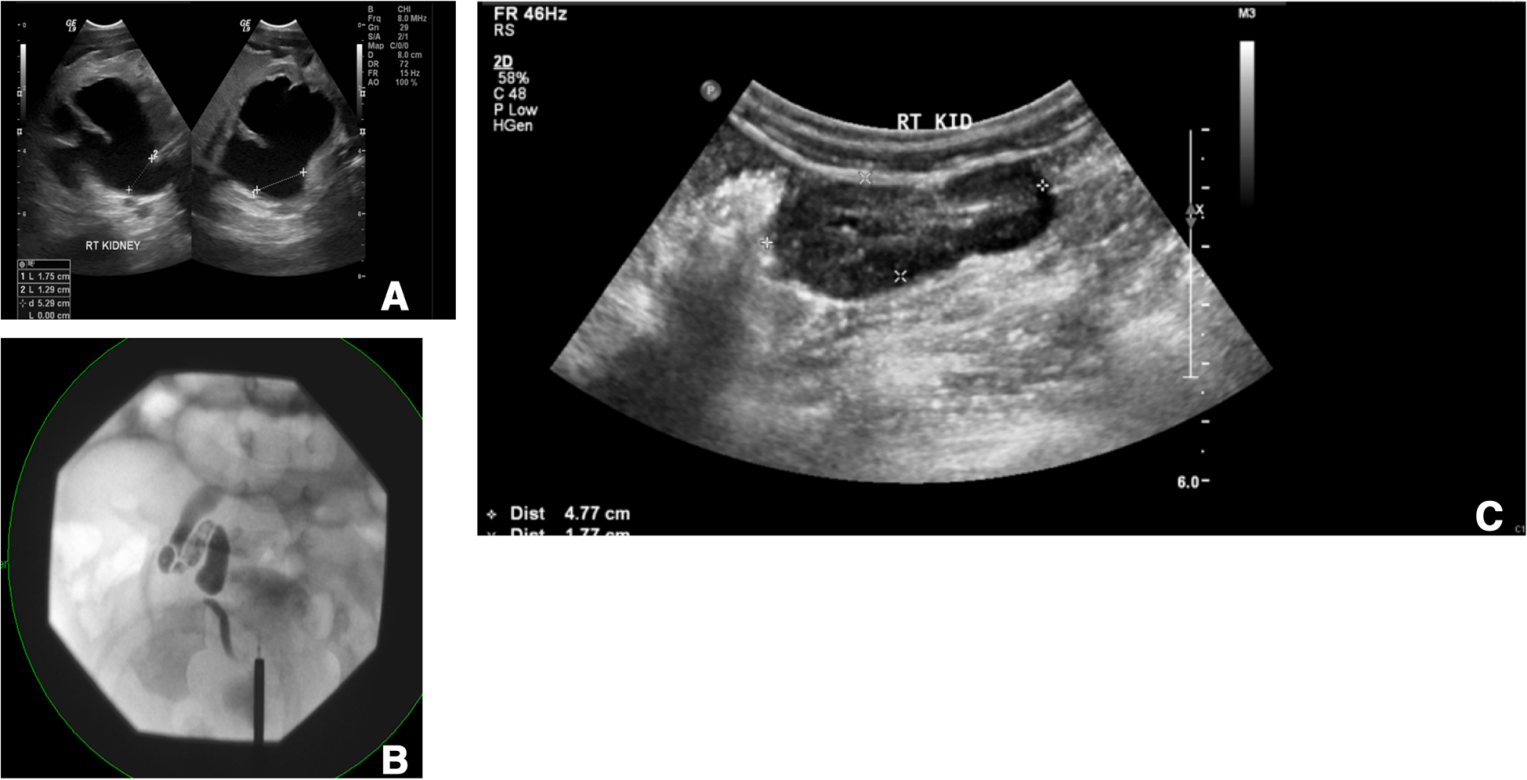
(**a**) Right renal ultrasound showed SFU grade 4 hydronephrosis. (**b**)Intraoperative retrograde pyelography showed right ureteral stricture at the level of the lower sacroiliac joint. (**c**)Renal ultrasound at 18 months postoperatively

At the age of one week, a male child presented with right antenatal hydronephrosis. His postnatal ultrasound showed Society for Fetal Urology (SFU) grade 4 hydronephrosis without clear hydroureter for the right kidney; the left kidney appeared to be normal (Fig. 1-a). A voiding cysto-urethrogram (VCUG) produced normal findings. Subsequently, a mercaptoacetyltriglycine (MAG3) renal scan at four weeks of age revealed a hydronephrotic obstructed right kidney (DRF = 46%) with poor washout in response to furosemide (T1/2 = 24 min). Accordingly, the patient was admitted electively and underwent right RGP (Fig. 1-b). Right mid-ureteral stricture was detected. The stenosed segment was resected (1.5*0.5 cm), and oblique ureteroureterostomy was performed. A pathological review revealed focal chronic ureteral inflammation with narrow lumen (1 mm). The most recent ultrasound, which was performed 18 months postoperatively, showed complete resolution of hydronephrosis (Fig. 1-c).

### Case 2

**Fig. 2:**
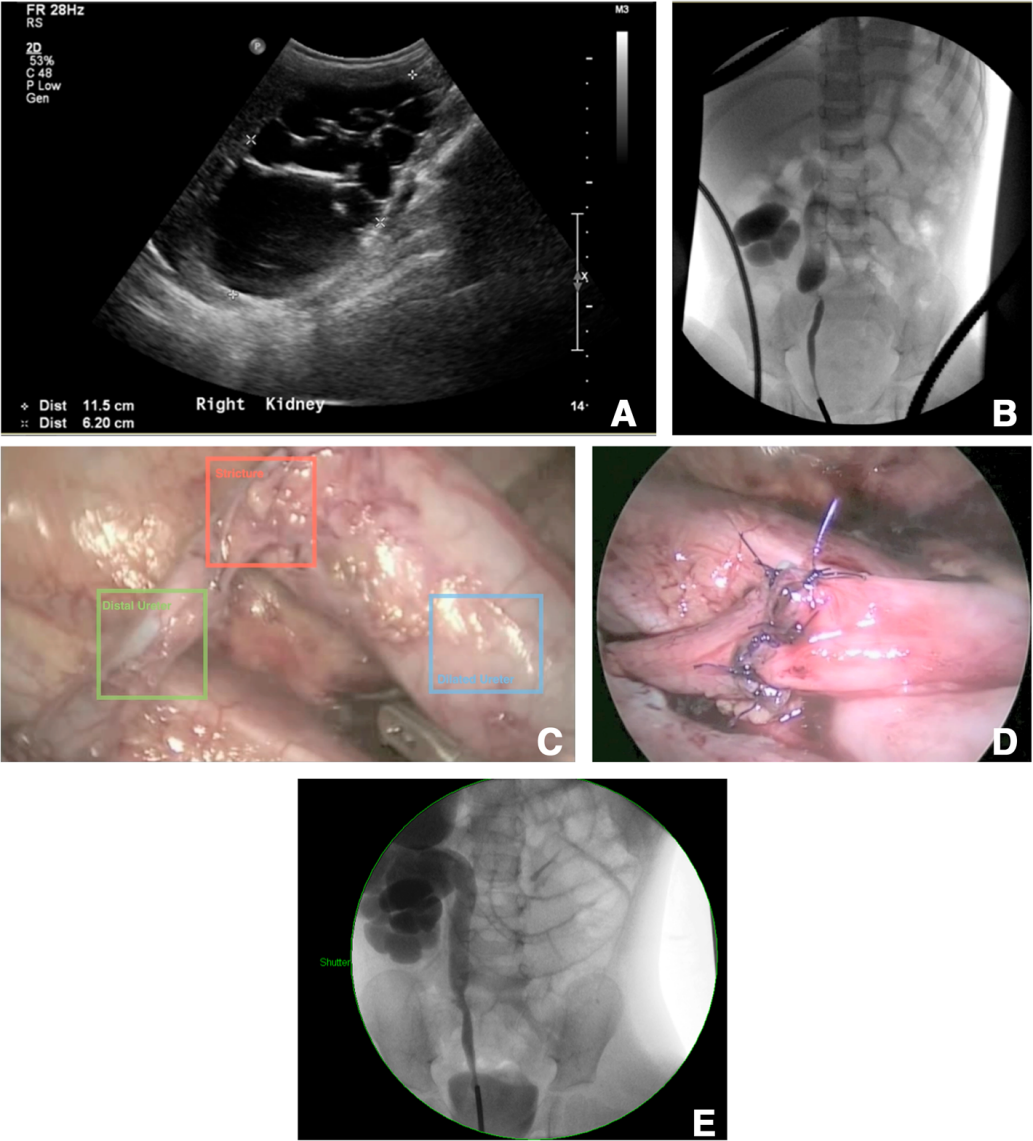
(**a**) Preoperative ultrasound showed SFU grade 4 hydronephrosis. (**b**) Retrograde pyelogram demonstrated right ureteral stricture at the level of the right mid-sacroiliac joint with proximal mega-ureter. (**c**) Laparoscopic view of the mid-ureteral stricture. (**d**) Image obtained at the end of the laparoscopic ureteroureterostomy. (**e**) Retrograde pyelogram showed a patent ureteral lumen after double-J stent removal

A two-year-old girl was referred to our institute due to incidentally discovered hydronephrosis. She was investigated for abdominal pain, and abdominal ultrasound revealed SFU grade 4 right hydronephrosis without clear hydroureter (Fig. 2-a). Initially, vesicoureteric reflux was excluded based on a normal VCUG. A MAG3 renal scan revealed a hydronephrotic right kidney with reduced global cortical uptake, no response to Lasix, and split renal function of 32% on the right side. The patient was admitted electively, and right RGP showed right mid-ureteral stricture with a length of 1 cm (Fig. 2-b). Subsequently, the patient underwent laparoscopic excision of the stricture segment and ureteroureterostomy (Fig. 2-c, d). A pathological report indicated predominant sever chronic inflammation with foreign body giant cell infiltration of the ureteral wall with severely stenosed lumen. Right RGP was performed at the time of stent removal and showed smooth passage of contrast media up to the pelvicalyceal system (Fig. 2-e). An ultrasound examination performed 30 months postoperatively revealed SFU grade 1 hydronephrosis.

## Discussion and conclusions

Mid-ureteral stricture is not a common cause of congenital hydronephrosis and is much less frequent than proximal or distal stricture [3]. Campbell published an autopsy series of 12 thousnads children. He found congenital ureteral obstruction in 1:150 autposies. Only 4% of them had a mid-ureteral obstruction. This outlines the rarity of CMS as a cause of congenital hydronephrosis [4].

Many theories have attempted to attribute stricture formation during embryogenesis to various causes, including a localized area of developmental arrest caused by extrinsic compression by foetal vessels during intrauterine life, a congenital ureteral valve, intrauterine ureteritis, and incomplete recanalization of the ureter [1, 5–8]. However, the exact explanation remains unclear. Mid-ureteral stricture may appear as a definite stricture or as a true valve without lumen stenosis [9]. The cases described here involved definite lumen stenosis, and no valves were detected.

CMS may be associated with other congenital renal anomalies, including crossed renal ectopia [2], multicystic dysplastic contralateral kidney [8, 10], solitary kidney [11], contralateral blind ending ureter [11, 12], and ectopic ureter of a duplex system [13]. However, our current cases exhibited no congenital renal anomalies other than ureteral stricture. CMS is mostly diagnosed as a unilateral disorder; however, cases with bilateral anomalies have been reported in the literature [14]. Our cases involved CMS on one side with an apparently normal contralateral side.

CMS is typically not diagnosed preoperatively, and definite diagnoses have been reached via retrograde assessment of the ureter [3, 14]. Burgnara et al. reported one case of CMS diagnosed using foetal MRI in which prenatal ultrasound showed progressive left hydronephrosis with suspected proximal left ureteral dilatation; this diagnosis was confirmed postnatally by intraoperative RGP [15]. RGP remains controversial as a routine preoperative imaging procedure in cases of congenital hydronephrosis. Routine preoperative RGP is recommended in cases involving a diagnosis of an unexpected ureteral lesion, such as mid-ureteral stricture, ureteral polyp and retrocaval ureter. In addition to confirming this diagnosis, RGP will allow for the proposed surgical intervention to be performed without requiring extension of the incision or anastomosis with inappropriate exposure [8]. For this reason, Hawang et al. recommended routine RGP prior to repair, during the same anaesthesia session, unless the ureter distal to the point of obstruction has been well visualized by other means [3]. Conversely, Rushton et al. did not recommend routine RGP based on findings from 108 pyeloplasties performed between 1986 and 1992, and they also found that RGP was not necessary for successful repair [16]. Both of the patients described in the present report were initially diagnosed using RGP. RGP was helpful for not only assessing stricture size and length but also ensuring appropriate decision making. We recommend routine preoperative RGP to avoid operator errors during ultrasound and the exclusion of unexpected ureteral lesions.

In our institution, postoperative renogram is only indicated if deterioration of hydronephrosis is observed in any of postoperative ultrasound scans or there is a poor renal function preoperatively. Both cases had improved hydronephrosis in consecutive postoperative renal ultrasound scans; thus, postoperative renal scan was no indicated. Moreover, the preoperative DRF of our included renal units was acceptable.

The management of CMS involves resection of the stricture and re-anastomosis of the ureter, with no role for conservative management [3]. Our definitive management, which was the same as that described in the literature, included resection of the stenotic area and re-anastomosis of the ureter. In our second case, this procedure was performed via transperitoneal laparoscopy.

In our cases, chronic inflammatory cells were predominant in the excised stricture segments. Hawang et al. reported the presence of inflammatory cells in the stenotic area, although these cells did not appear to be significant. In their study, they found asymmetry in the thickness of the muscularis mucosa with non-significan acute or chronic inflammation of the stenosed segement in some cases [3]. In our second case, sever chronic inflmmation was clearly observed and there was a focal inflammation in case 1. This may refelct the role of inflammation in some cases of CMS.

Postoperative long-term follow-up revealed the regression of hydronephrosis. In most of the relevant literature, improvement in hydronephrosis and promising renal function have been reported during short-term follow-up [2, 10, 16].

CMS is a rare cause of congenital hydronephrosis that should be considered whenever proximal mega-ureter is an associated finding. Despite advanced radiological modalities, RGP remains the mainstay approach for diagnosing ureteral anomalies.

## Abbreviation

CMS: Congenital mid-ureteral stricture
MAG-3: Mercaptoacetyltriglycine
RGP: Retrograde pyelography
SFU: Society for Fetal Urology
VCUG: Voiding cysto-urethrogram
